# Participatory development and implementation of inclusive digital health communication on COVID-19 with homeless people

**DOI:** 10.3389/fpubh.2022.1042677

**Published:** 2022-11-10

**Authors:** Anabell Specht, Navina Sarma, Tabea Linzbach, Theresa Hellmund, Merle Hörig, Mia Wintel, Gabriela Equihua Martinez, Joachim Seybold, Andreas K. Lindner

**Affiliations:** ^1^Charité – Universitätsmedizin Berlin, Corporate Member of Freie Universität Berlin and Humboldt-Universität zu Berlin, Institute of Tropical Medicine and International Health, Berlin, Germany; ^2^Department of Infectious Disease Epidemiology, Robert Koch Institute, Berlin, Germany; ^3^Charité – Universitätsmedizin Berlin, Corporate Member of Freie Universität Berlin and Humboldt-Universität zu Berlin, Medical Directorate, Berlin, Germany

**Keywords:** homeless, participatory research (PR), poverty and inequality, health communication, digital gap, SARS-CoV-2, COVID-19

## Abstract

**Introduction:**

People experiencing homelessness (PEH) are disproportionally affected by the COVID-19 pandemic. The realities of their daily lives have been given little consideration in the pandemic response. They are not represented in existing health information campaigns, and many are structurally excluded from digital information. The project aimed to develop inclusive COVID-19-information material to strengthen infection prevention and control of PEH.

**Material and methods:**

In a participatory process, PEH were involved in the planning, production, and evaluation of poster and video information material on COVID-19. Various stakeholders were consulted for external supervision. Service providers all over Germany were informed about the material that could be ordered free of charge. For the evaluation, semi-structured interviews with homeless service providers and PEH were conducted, and the online views of the videos were measured.

**Results:**

Sixteen PEH participated actively in the project. Two COVID-19-information videos were launched in 5 languages in February 2021. Posters promoting vaccination against COVID-19 were produced in 9 languages. As of May 2022, the videos have been viewed more than 2,000 times. A total of 163 service providers for PEH and public institutions received the posters, thereof 72 upon request. Twelve service providers and 8 PEH participated in the evaluation. They pointed out the lack of targeted information material for PEH. The consideration of the concerns and the diverse representation of PEH was perceived as particularly important. Most of the service providers were unable to show the videos due to technical and spatial limitations. Digital challenges for PEH, like the lack of and maintenance of a smart phone, became apparent.

**Conclusion:**

The cooperation of research, practice and the community were key factors for the realization of this project. Strong links to the community and the involvement of relevant stakeholders are indispensable when working with PEH. Exclusion from digital information is an increasingly important component of the structural marginalization of PEH. Digital inclusion for PEH and service providers can help to counteract social and health inequalities. The lessons learned through this project can contribute to strengthen participation of PEH and to consider their perspectives in future health communication strategies.

## Introduction

People experiencing homelessness (PEH) are disproportionally affected by the COVID-19 pandemic. Precarious living conditions on the street, in encampments and cramped shelters, limited access to hygienic supplies and prevention measures, stigmatization, marginalization from social, political and economic resources as well as exclusion from health services result in high rates of underlying health conditions and a high risk of SARS-CoV-2-infection (1–3). The prevalence among homeless individuals may be similar to that found in the general population, however, the increased risk of outbreaks with high infections rates has to be considered (4). Also, social determinants and pre-existing health conditions place PEH at higher risk of severe COVID-19 infection (5, 6). However, studies to properly assess the outcome of SARS-CoV-2 infection in PEH are still required (3).

Since the beginning of the COVID-19 pandemic, the need to consider the living conditions of PEH when implementing measures of infection control and prevention (IPC) has been addressed by the German national working group on homelessness services (Bundesarbeitsgemeinschaft Wohnungslosenhilfe, BAG W) (7). In July 2021, the Robert Koch Institute, the national public health institute in Germany, published targeted recommendations for COVID-19 in the context of homelessness that were developed together with experts from the field (8). Many good practice solutions were implemented locally to protect PEH during the pandemic, such as provision of adequate isolation and quarantine options addressing possible complex needs of PEH, 24/7 accommodation with single rooms, regular voluntary universal testing for SARS-CoV-2, and mobile vaccination campaigns (9, 10). However, in many places, isolation, access to vaccination and testing as well as targeted information remained a challenge. This is particularly critical because it is known from past respiratory viral outbreaks that these control measures are crucial in managing epidemics or pandemics (11). In our opinion, PEH in Germany are to date not sufficiently addressed in the pandemic response including information campaigns.

With the onset of the pandemic, digitalization was discussed more publicly than before as it affected everyone to a substantial extent. Being digitalized took on a new relevance, as it was, for example, a prerequisite for the digital EU certificate, which allowed unrestricted access to public buildings, use of transport and freedom to travel between international borders (12). Multiple problems faced by PEH result from their precarious socioeconomic situation which also affects the ability to maintain a digital device and to have access to internet-based services (13). Digital inequalities result in further social exclusion as it limits career opportunities, represents a barrier to maintaining social and service-related contacts, causes financial hardship and are a determinant of health (14–16). At the same time, digitalization can be an opportunity for better social inclusion (14, 17).

In a previous COVID-19 project among PEH, we identified the necessity to address language barriers, to include digital information formats, and to use participatory approaches considering homeless people's needs and life situation (9). This follow-up project aimed a participatory development of inclusive health communication on COVID-19 to strengthen options for IPC for PEH. We describe the development, implementation, and evaluation of targeted digital as well as non-digital (hybrid) health communication material (videos and posters).

## Materials and methods

This project was conducted over a period of 11 months, from October 2020 to August 2021 in Berlin, Germany. According to the principle that in participatory projects participants should benefit directly from the research process (18), the perspective of PEH represented the basis for all project steps, and all PEH had decision-making power and were paid for their work effort.

### Study team and recruitment of community partners

**Figure 1:**
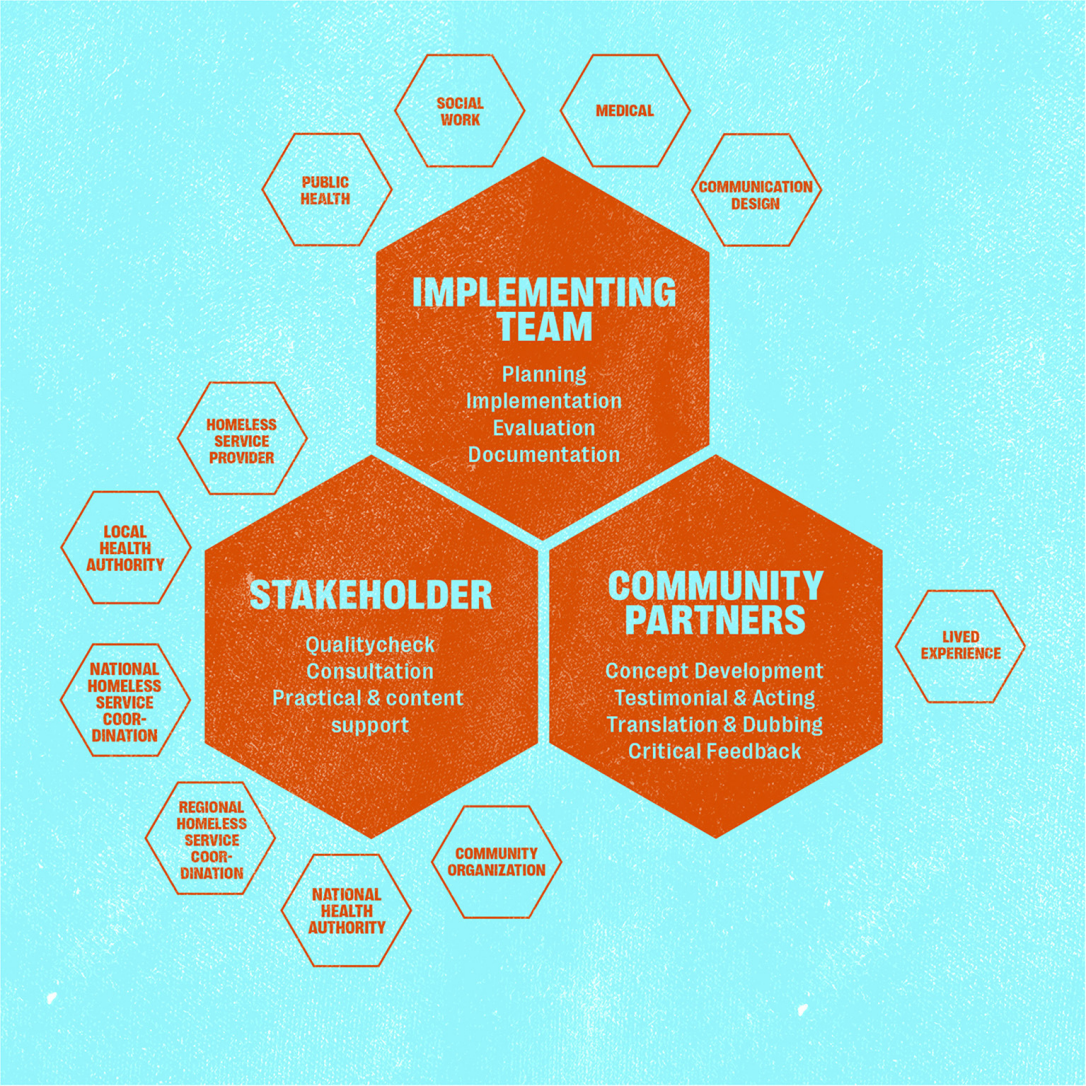
Presentation of study team with expertise and responsibilities, including people experiencing homelessness as community partners and various stakeholders.

The study was initiated and supervised by two experts from the fields of medicine and public health. The study team (Figure 1) also included a clinician, a health scientist, two social workers, two student assistants and a professional communications designer. Four of the team members had long-term work experience with PEH. The PEH who participated as community partners were actively involved in the planning, production, evaluation and decision-making processes. They were the protagonists with one main actor for the videos and 12 further actors for portraits (vignettes) in the videos and posters. Three PEH had reservations to be filmed or photographed, and were involved in translations, sound recordings or evaluation rounds. Community partners were recruited in a social facility for PEH operated by the Berliner Stadtmission in Berlin. The facility included a 24/7 shelter, a medical outpatient clinic and a clothing store, where people could easily be approached during the day and in the waiting areas. Some members of the study team were already known to the PEH from their (voluntary) work at this facility, which formed the trust base to approach people directly. We showed a short film clip (mood board) to the PEH in order to introduce them to the project. The communication designer created the clip especially for recruitment purposes. Inclusion criteria were age above 18 years, current or previous homelessness and written informed consent. We aimed at partnering with PEH from different age groups, genders, (dis-)abilities, languages and country of origin to reflect the diverse image of PEH in Berlin and to create material that is easier to identify with.

We consulted the following stakeholders for external supervision: the German national working group on homelessness services (BAG W), a self-organization (Selbstvertretung wohnungsloser Menschen e. V.), the coordination group for homeless shelters in Berlin (Koordinierungsstelle Berliner Kältehilfe), the Robert Koch Institute as well as staff of the Berliner Stadtmission (Figure 1).

### Videos

Scripts in German language were created for two videos with general information about COVID-19 and SARS-CoV-2 testing. Expertise of the medical specialists and social workers and the experience of the community partners were combined to gain a better understanding of the health, personal needs and coping strategies of PEH during the pandemic. The scripts were edited in a multi-step revision process. After they were shared with and adapted by all stakeholders, the main protagonist translated them to a simple and clear language. Native speakers translated the scripts into four other languages that were identified in the previous project we performed (9). Quality control was performed by professional translators. The main actor speaks German in the videos. Three PEH and one professional actor have dubbed the clips into Russian, Polish, Romanian and English.

### Posters

Information posters with precise key messages on access to vaccination were developed and provided in a digital and printed version to support the public COVID-19 vaccination campaign for PEH that started in Berlin in March 2021. To identify main questions or concerns of PEH regarding vaccinations, we consulted PEH and staff of service providers, as well as our stakeholders.

### Participation and participatory decision making

The shooting locations were chosen together with the protagonists. The aim was to find locations that would create a pleasant working environment and at the same time provide images that PEH could identify with and which would not reproduce stereotypes. For the whole process, the individual needs of the protagonists were considered. This included for example a transport service if needed, food and drinks, as well as access to barrier-free sanitary facilities at all locations.

The selection and editing of the video material and photo motifs took place during several feedback rounds with community partners. The videos were watched together several times on a screen in the common room of the homeless shelter. Attending community partners and other PEH were asked for their opinion and criticism, e.g., about the content and the locations. Stakeholders who were not directly involved in the production were also asked for feedback.

### Dissemination of the information material

On April 2021, a website was created in order to provide open access to the videos and posters, as well as to provide further information about the project (19). Furthermore, the material was disseminated through various social media channels (Twitter, Facebook and Instagram of the participating institutions). The printed posters were sent to all homeless service providers that were part of the Kältehilfe Network in Berlin (shelters, soup kitchens, warm rooms, medical facilities for people without health insurance and counseling centers). The German national working group on homelessness services informed service providers all over Germany through their network about the project and the offer to order the posters free of charge.

### Evaluation and data analysis

All institutions that received material were listed. A telephone survey with randomly selected homeless service providers who had received the printed posters was conducted. Recruitment for the survey took place *via* email. After written informed consent, a semi-structured telephone interview (Supplementary Interview Guideline 1) was conducted for 15 min addressing the practical implementation and perception of the posters and the videos.

A social worker interviewed PEH in two shelters of the Berliner Stadtmission to determine how the material was perceived by PEH. Participants were randomly approached during the service hours of the shelters. After written informed consent, a semi-structured interview (Supplementary Interview Guideline 2) was conducted for 20 min.

The data analysis was based on written notes taken during the qualitative phone interviews and face to face interviews. A systematic qualitative content analysis was undertaken (20). After reviewing the data material, it was coded by an inductive procedure and summarized in categories.

Furthermore, the usage of the project website with the number of video views was measured.

### Ethics

This study was approved by the Ethics Committee of the Charité – Universitätsmedizin Berlin (No.: EA2/168/21). All PEH who contributed to the implementation of the project were paid for their work. The study was explained to PEH in the preferred language, and written informed consent was provided for participation. The scope and time frame of the project were transparently communicated, as well as the possibility to withdraw participation at any point of the project without repercussions. As PEH are a particularly vulnerable group, privacy, data security and a familiar atmosphere (to avoid any discomfort) were taken into careful consideration during the interviews.

## Results

### Participatory production

Sixteen PEH participated actively in the production of the information material. All participants were experiencing homelessness at the time of the study. Ten protagonists were recruited in the 24/7 shelter, 3 in the clothing store and 3 were recruited in the streets during the shooting days. Four of the participants were women and 12 were men, aged between 25 and 75 years from 6 countries speaking 8 different languages, two of them were in a wheelchair.

We produced two multilingual COVID-19 information videos under the slogan “We keep Corona off the streets”. The protagonist changed some parts of the script for a better understanding. For example, regarding information about the opioid substitution programme in the quarantine facility, “*substitution is provided*” was replaced by “*you can stay there, also if you consume*”. The 5-day shooting of the videos took place at 15 locations.

The videos were produced with 13 protagonists. Six protagonists chose spots on the grounds of the Berliner Stadtmission or in the near vicinity as shooting locations, while 7 chose spots around the main railway station, a nearby park and public places.

The first video clip (duration 3 min 13 s) contained general information about COVID-19 (Supplementary Video S1). It explained the transmission modes of SARS-CoV-2, symptoms, increased risk of infection among PEH and strategies for self-protection (hygiene measures). The second video clip (duration 1 min 14 s) contained details about COVID-19 testing and the proceeding after a positive test result for PEH who lack the possibility of self-isolation at home (Supplementary Video S2). Thus, the video talks about the possibility of isolation in quarantine accommodations that consider the needs of PEH in a sensitive way.

**Figure 2:**
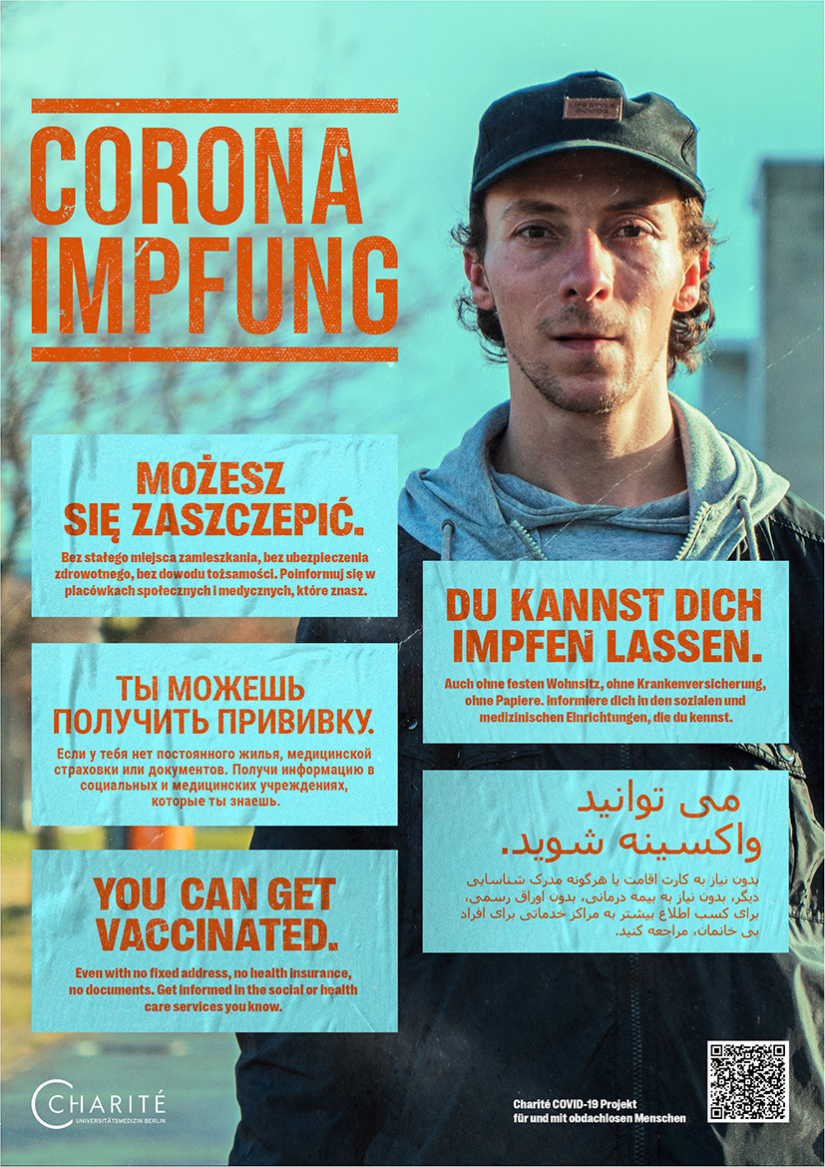
Multilingual poster disseminated digitally and in print to support the public COVID-19 vaccination campaign for people experiencing homelessness (one out of seven different motifs).

**Figure 3:**
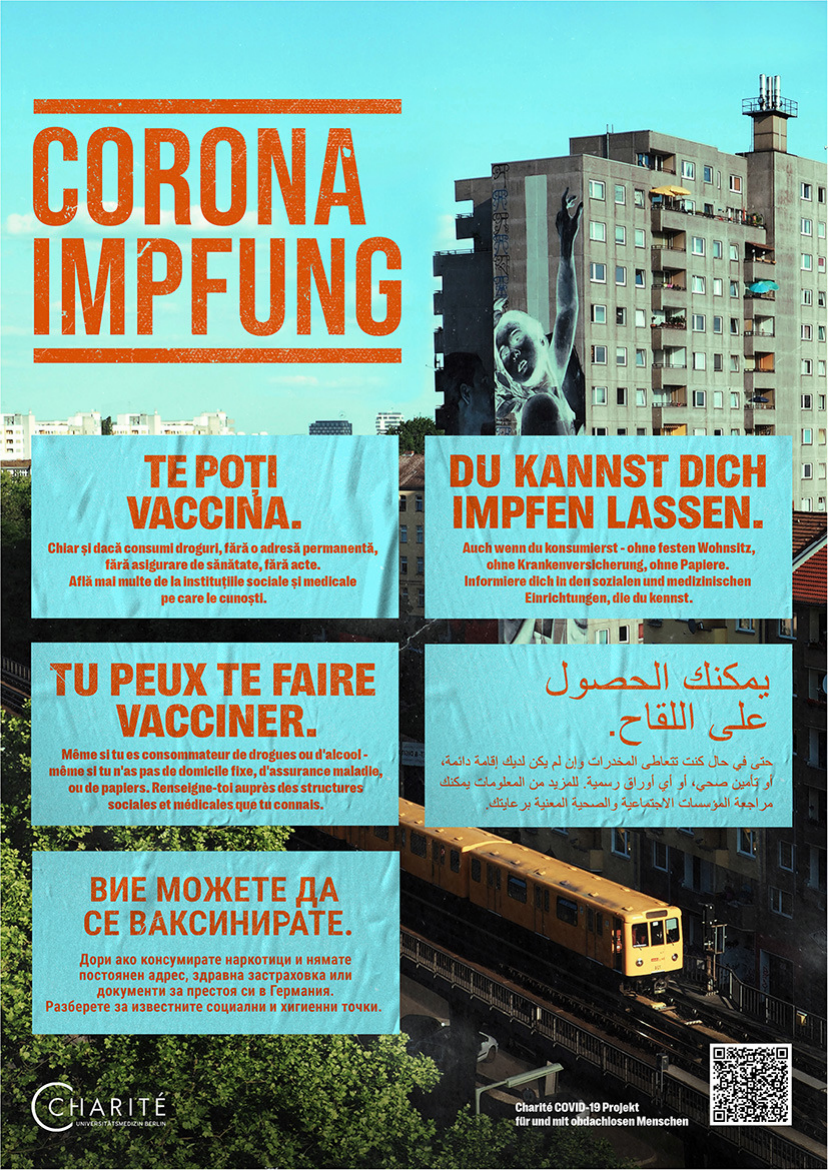
Multilingual poster disseminated digitally and in print to support the public COVID-19 vaccination campaign for people experiencing homelessness, which also addresses drug use (one out of seven different motifs).

In the second step, we designed multilingual posters with seven different motifs (Figures 2, 3) (19). With two versions, we have covered a total of nine languages identified as most relevant in a previous local project (9):

- 1st language version: German, Polish, English, Farsi, Russian
- 2nd language version: German, Romanian, Bulgarian, Arabic, French

Out of the 13 protagonists in the videos, three participated in the production of the posters. Two more were recruited in the 24/7 shelter. Portraits were taken on two shooting days. The main questions or uncertainties about the SARS-CoV-2 vaccination among PEH were identified through discussions with our community partners and staff of homeless service providers. These were having access to vaccination without a permanent address, documents or health insurance, as well as implications of drug use. Accordingly, the posters were designed with the slogan “*You can get vaccinated. Even with no fixed address, no health insurance, no documents. Get informed in the social or health care services you know*”. In another poster version we included the sentence “*Even if you use drugs*”. The posters that address drug use do not contain portraits of PEH to avoid stigmatization (Figure 3). As a result of the feedback rounds, the hybrid nature of the posters was extended by including a QR-code linking the poster with the project website.

### Dissemination

The videos were launched on February 11, 2021 during a hybrid (online and in presence) event. It was screened in the homeless shelter of Berliner Stadtmission under COVID-19 hygiene measures to allow community partners without internet access or mobile devices to participate. Over 100 participants joined online from various fields such as homeless services, politics, research and community.

**Figure 4:**
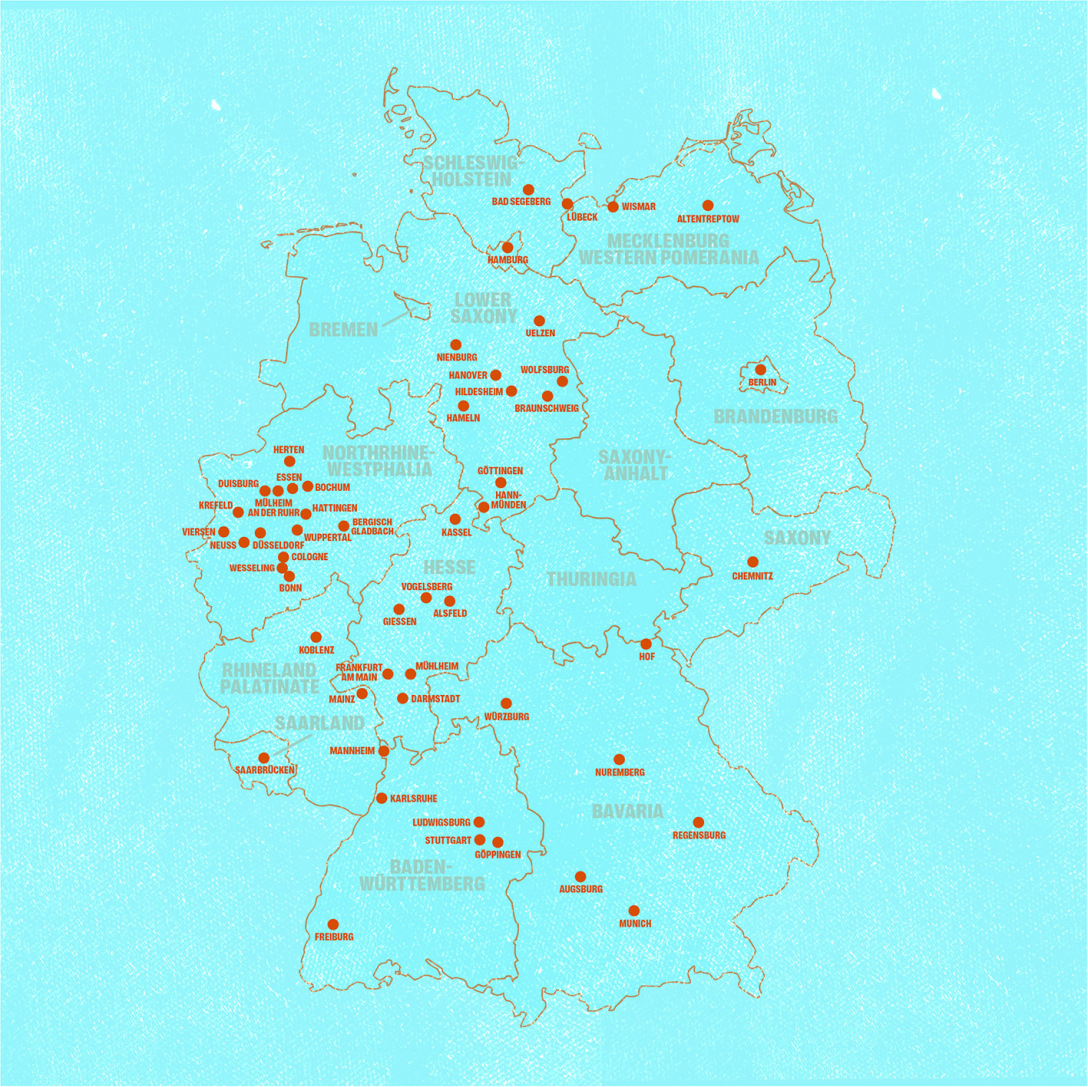
Map of Germany showing the cities where institution received the printed posters without or with request. From February 2021 until May 2022, a total of 1.754 posters were sent to 163 institutions in 53 cities.

By May 2022, a total of 1,754 posters were sent to 163 institutions and facilities in 53 cities within Germany (Figure 4; Supplementary Table S1). Whereas a set of posters was automatically sent out to all 91 institutions that were part of the Berlin Kältehilfe list, the others (72) received the material upon request. The institutions included facilities for PEH such as clothing facilities, day care centers, consulting services, hygiene facilities, medical facilities and night shelters (146), facilities for drug users (9), facilities for refugees (2) and municipalities and public facilities such as a library and public authorities (6). The distribution period lasted from February 2021 until May 2022. The dissemination *via* the social media channel of the Charité – Universitätsmedizin Berlin took place in February 2021. Between February 8, 2021 and May 31, 2022, the videos have been viewed 2,064 times *via* the project website. We registered peaks in the numbers of requests of the posters at the beginning of the vaccination campaigns in spring 2021 and then again in winter 2021. At that time, the booster vaccinations started, the night shelters opened for winter season, and the institutions were informed about the material by email.

### Evaluation

Out of 20 homeless service providers that were invited to the evaluation, 12 agreed to participate in a telephone interview which were conducted by two of our study team members in August 2021. At the same time, another team member interviewed 8 PEH on 2 days in front of a shelter and in a day center. All were experiencing homelessness at the time of the study. Out of the notes that were taken during the interviews, thirteen categories of different topics were identified (Tables 1, 2). Not all categories were addressed by everyone. There was a general appreciation of how the information was presented in terms of acceptability and sensitivity toward the targeted population.

**Table 1:** Evaluation of the videos and posters according to semi-structured telephone interviews with 12 institutions.

	**Achievements**	**Challenges**	**Recommendations**
1. Feedback on the material (posters and videos) in general	Information reached the stakeholders *via* different ways: • Berlin Kältehilfe list (5) • online search (2) • homeless service providers (2) • source unknown (3) Diverse and barrier-free (analog, hybrid, and digital) material was appreciated (12) Brochures and flyers continue to be popular in respondents' workplace. Regardless of the type of materials, importance was placed on: • a simple, clear, and concise message • purposeful design • multilingualism	Lack of targeted information material on health topics in general	To reach a larger population, materials should be distributed *via* diverse ways (e.g., mailing lists) Use diverse information modalities and combination of those to reach heterogeneous group (2) Need of similarly tailored information material on diverse (health) topics for daily work with PEH such as: personal hygiene (e.g., showering facilities) (2), preventive medical check-ups (2), medical care options also for people without health insurance or documents (2), scabies treatment, low-threshold psychiatric services, hepatitis (2), sexually transmitted infections, standard vaccinations, counseling on health insurance, safer drug use, tuberculosis, HIV, cancer prevention, and access to health services in general
2. Feedback on the vaccination posters concerning use and benefits^a^	All interview partners used the posters in locations for PEH (12) Positive aspects that were mentioned: • appropriate for the counseling context • multilingualism (4) • professional design (2) • diversity-sensitive (2) • simple and clear language, suitable for functional illiterates • accepting drug policy (2) • opened the conversation on vaccination and enabled further counseling (7) • initiated conversation about taboo topics (e.g., drug consumption) (3) • enabled PEH to inform themselves ‘quietly' in their preferred language (5) • informed PEH that they had the right to receive vaccinations	It was perceived as suboptimal that posters did not contain specific information on local vaccination campaign (e.g., timing and places) (2) People have scanned the QR code to the homepage in the false assumption that they can make an appointment for vaccination (3) Posters were removed several times (without obvious vandalism) (2)	The QR-Code could link to further information on vaccinations and facilitate appointments for vaccination. Distinct local information on vaccination programmes was added directly on the posters (2) The following aspects could be considered in the posters: • poster version for those who are undecided about vaccination (with a link to a hotline or low-threshold counseling) • clear reasons/arguments pro vaccination • clarification about fake news and vaccination myths • more diversity for example in terms of women Posters can be placed in different locations such as: • outpatient clinic buses • shelters (3) • consulting sites (2) • restrooms (2) • train stations (2) • community welfare centers (2) • soup kitchens (2)
3. Feedback on the videos concerning use and benefits^b^	Videos were utilized in the counseling context	“*I watched the general video and thought it was great. So it's even more unfortunate that I immediately asked myself, how are we going to use this in our service because it's not actually feasible.”*	
		Reasons for not using the videos: • lack of time • lack of staff • lack of equipment (screens, accessible PCs or tablets, loudspeakers) (5) • rooms are often too crowded and restless (2) PEH who have a smartphone might have only limited mobile data available, which makes streaming of videos difficult (2)	
4. Opinions about the use of posters for health information	Getting predesigned material is helpful Posters can initiate the conversation on specific topics Posters are a mean to spread important information in an unobtrusive way	The institutions do not have the resources to design information materials themselves Excessive information material in the facilities is overwhelming	Health-information posters should have concise information content PEH sometimes lack the conversation with people in everyday life. Social workers or medical staff can be confidants, especially when no other social network is available (2) “*Individual personal contact is irreplaceable”*
5. Digitalization in general	PEH that are from a younger generation attach more importance to owning a smartphone and have a greater affinity for digital media Digital material as an opportunity to reach PEH, also people experiencing hidden homelessness Helpful in counseling contexts (5)	Gap in digitalization on both sides, within the institutions and among PEH (8) Lack of appropriate equipment. Personnel, space and financial capacities are limited therefore videos could not be screened (8) Reasons why PEH are excluded from digitalization: • expenses implied • difficult in recharging the phone / digital devices • loss of device • stolen device due to lack of access to safe storage • when a smartphone is available, the volume of mobile data is limited and used sparingly	Audible material can be helpful for visually impaired people Situations and contexts in which digitalization can be useful • questionnaires • (anonymous) online counseling • interpreter service (via video call)

The answers were structured in categories commenting on achievements, challenges, and recommendations. In the case of multiple mentions of a specific aspect, the number of mentions is given in brackets.

a All 12 participants were familiar with the posters before the interview.

b Six out of 12 participants were familiar with the videos before the interview.

**Table 2:** Evaluation of the videos and posters according to semi-structured interviews with 8 people experiencing homelessness (PEH).

	**Positive aspects**	**Negative aspects**	**Recommendations**
1. General feedback on the posters and videos	Peer-approach is great Videos and posters were both well accepted (2) Visualized information was seen as beneficial, particularly for illiterates (3)		Use written media, such as newspapers, for distribution, as they are used as a source of information (3) Videos are more helpful because they are visually and aurally appealing (3)
2. Where did PEH see the posters?^a^	Homeless facility (2) Homeless shelter Outpatient clinic Restroom Railway station		Posters could be placed in - public transport - churches - public information boards
3. Design of the vaccination posters	Participants liked the posters (7), because: • statements perceived as clear and visible (4) • variety of languages used • people feel addressed/can identify (5) Positive aspects on the choice of motifs: • underprivileged people are the protagonists • individual portraits (2) • variety of motifs (2)	Motifs with PEH or urban motifs do not appeal to everyone because it can remind people of their own harsh realities (2) Excessive text Unsuited for illiterates (3)	The following aspects could be considered in the posters: • also depict drug consumers • utilize city landmarks for identification (2) • portray more women • portray disabilities more obviously • use of one language per poster is clearer • show more positive motives, for example nature
4. Use and benefits of the vaccination posters	Informative (5) Inspiration to think/speak about vaccination/COVID-19, but no influence on the decision to get vaccinated (3)	Not useful Risk of going unnoticed	
5. Design of the videos^b^	The videos were appreciated (8) and perceived as: • concise (3) • easy to understand (5) People felt addressed (4) and could identify: • with the protagonists (2) • with the speaker (4) • with the diversity of protagonists • due to the realistic representation *"It's the reality”*		Suggestions to improve the videos: • voice could be livelier • to depict individuals who take drugs • to provides Turkish and Arabic translation • key messages conveyed at the beginning of the videos
6. Use and benefits of the videos	Perception of videos being an effective mean for health information distribution for those who possess a mobile phone (4) The information about the possibility of getting vaccinated even without health insurance and in case of drug use was perceived as particularly useful		Distribution of flat rates to the PEH during the pandemic (7)
7. Other information/questions that came up during the interviews	Test site refused rapid antigen testing due to lack of identification Question if the isolation ward is still in place COVID-19 vaccination was well tolerated despite drug use		

The answers were structured in categories commenting on positive and negative aspects as well as recommendations. In the case of multiple mentions of a specific aspect, the number of mentions is given in brackets.

a Three of the 8 participants had seen the posters before.

b None of the participants had seen the videos before the interview.

All service providers approved that the posters were widely used for the vaccination campaigns. All 3 respondents who knew the videos considered them helpful for providing targeted information and allowing PEH to feel addressed directly. However, the streaming of the videos in the services was reported to be difficult due to lack of digital devices. Only 1 institution reported to have used the videos within the counseling context. Eight out of 12 service providers pointed out the digital challenges for PEH, e.g., owning and maintaining a mobile or smart phone, and the lacking digital infrastructure in services for PEH. It was acknowledged that digital tools and offers can be a chance to provide better health and social care for PEH, also those living in hidden homelessness. All respondents spoke about the lack of targeted information material for PEH in general. It was suggested to address a wide range of further topics with targeted digital information material.

Out of 8 PEH, 3 had seen the posters in relevant facilities of homelessness services in Berlin. None had seen the videos before. Participants appreciated that PEH were protagonists of the videos and posters, but even more diversity would have been appreciated. They could identify with the material and pointed out that mentioning consuming drugs and alcohol was crucial. It was reported that the posters encouraged PEH to think about a vaccination. Precise information on vaccination offers in the respective locations would have been helpful. Both, dissemination of information through posters and videos was appreciated. It was pointed out, that visually and auditorily appealing videos could be suitable especially for illiterate people. Respondents appreciated the idea of disseminating information *via* videos. One person addressed the digital gap, saying that it was only useful if you had the convenience of owning a digital device.

## Discussion

### The participatory approach involves all levels of knowledge

A key factor for the realization of the videos and posters was the set-up of an interdisciplinary team together with community partners and thus bridging the gap between research, practice and community. Of particular significance were good contacts of the study team with homeless service providers and PEH. Furthermore, it was crucial to involve various local and national stakeholders right from the beginning of the project for advice, support and distribution of the materials. The participatory approach enables active generation of knowledge together with practice and communities (21). Accordingly, we addressed hierarchies transparently and valued PEH's own life experiences as equivalent to knowledge from professionals within the fields of social work and public health.

A challenge was to deal with poverty and precarious living conditions in a sensitive way. Poverty was neither to be tabooed nor trivialized. The goal was to show a realistic picture of the social system and living environment of PEH without reproducing stereotypes or exposing people. The image and portrayal of homelessness in times of the pandemic was therefore to be determined primarily by the community partners themselves. The main protagonist decided to participate by stating “*I think I'm someone people accept*”. In front of the camera, most PEH placed emphasis on a proud attitude showing an active and upright posture.

For the evaluation, 8 PEH and 12 service providers were interviewed. They acknowledged the sensitive implementation of the project. The consideration of the concerns and the diverse presentation of PEH was perceived as particularly important.

In this manuscript we have chosen the term “people experiencing homelessness” because it presents homelessness as one aspect among others and is less generalizing than the term “homeless people”.

### Participation and its limitations

To enable participation according to the needs of the community partners, work conditions were defined and arranged together. For example, the main protagonist's condition for participation was the assurance of permanent access to barrier-free sanitary facilities. He knew from personal experience that this would be a main challenge, especially during the pandemic when sanitary facilities were even less available for PEH. This highlights one of the fundamental problems that people living in the streets face on a daily basis.

For some people there were barriers that made participation difficult or impossible such as hidden homelessness, illegalization, and precarious employment. We have tried to enable safe participation for them as well. For some, a solution was to participate by dubbing the videos, translating the scripts or by attending the feedback sessions. Others chose to not participate altogether. Our impression was that it has been easier for people to participate if there was pre-existing contact with project staff or other PEH who had already taken part. In regard to the evaluation, we found it easier to find interview partners in the day center which provided a safe setting for the interview compared to the recruitment in front of the shelter where the interviews were performed outdoors. It is crucial to be aware of and respect the right to non-participation.

We did not collect socio demographic data from the PEH who participated in the production and evaluation of the materials. All were experiencing homelessness at the time of the study. Some had shelter available. Similar to varying infection risks, differences in the participation and responses between sheltered and unsheltered individuals are possible, but this could be not further analyzed in this study (22).

### Impact of the information material

Despite the mainly positive feedback in the evaluation, the impact of the videos could only be verified to a limited extent. Most of the homeless service providers were unable to show the videos on their premises, due to technical and spatial limitations. The videos and posters were widely distributed *via* social media and email lists. It remains unclear to what extent they reached the actual target group. However, considering the important role that social media has in shaping people's state of information and attitudes toward public health interventions such as vaccination campaigns, efforts should be increased to utilize these means of communication (23). The posters seem to have been particularly useful for the vaccination campaign according to the feedback of service providers and the number of poster-orders throughout Germany. Addressing the specific questions and concerns of PEH—e.g., having access to vaccination without a permanent address, documents or health insurance, as well as implications of drug use—may be one approach to increase vaccination coverage (24, 25).

The type of institutions that ordered the posters reflect the broad range of services that address the complex needs of PEH (26). Among them were services targeting people without official documents (e.g., passport, citizenship, health insurance), people living in poverty, people using drugs, as well as services exclusively provided for women. The way how people are approached as well as the information that is provided must be tailored to PEH's situation and needs. The videos and posters of this project demonstrate a step into this direction.

A concise point that was emphasized by staff in homeless service providers is that no information material can replace personal contact and face-to-face conversation. Relationships to social workers and medical staff remain essential—especially when people are in precarious situations, access to information is difficult and social contacts are limited.

### Limitations of the information material

In the evaluation, it was critically mentioned that the material provided only general information without any details on how to get access to vaccinations. As the provision of tests and vaccinations were locally organized in various different ways, specific information could not be integrated into the material. Some institutions therefore added distinct information for the local context directly on the posters.

The material intended to encourage testing and vaccination. One of the statements was that both is possible despite alcohol, other substance usage, and without official documents. Although this information was in line with official regulations, we could not guarantee appropriate implementation. In fact, reports from homeless service providers and PEH showed that people lacking identification documents had difficulties accessing public COVID-19 test and vaccination centers (27).

The material also stated that people with positive test results would be cared for according to their needs. However, it was frequently reported to us that quarantine and isolation capacities for PEH were insufficient, and that substitution or medical care was only partially offered.

### Benefit and challenges of digital inclusion among PEH

During the pandemic, interpersonal contact became limited and digitalization crucial. Most of the population has been able to continuously access updated information about the pandemic, whereas many PEH were excluded from this information flow due to technical and socio-economic reasons that make it difficult to acquire or maintain digital devices. Even with an internet-enabled device, access to information remains difficult with a lack of free Wi-Fi and a limited access to electricity. When fighting a pandemic, it is important to reach all people in society equally, albeit through different channels and in different languages. To ensure this, digitalization has to be facilitated for social and health institutions as well as for PEH (17, 28).

The digital gap also had to be considered in the planning and implementation of our project. Our community partners without mobile- or smartphones were visited personally by our study team for recruitment and further collaboration. People who were temporarily sheltered in a 24/7 facility were easier to locate than people living on the streets. Creative and individual ways had to be identified to stay in contact with all community partners, regardless of their housing or digital situation. This shows the daily challenges of PEH without digital access, that also affects the interaction with friends and relatives, employers, social and medical service providers as well as with public institutions.

However, it has to be noted that PEH are not per se excluded from digitalization. A considerable number of PEH manage to purchase and maintain a mobile phone or smartphone and find ways of charging it and accessing internet.

### Progressive health communication

There was a wide range of institutions that ordered the elaborated posters, including a library, facilities for people who use drugs as well as different facilities for specific populations. This demonstrates that PEH can and need to be reached in different ways that consider their complex needs and situations including hidden homelessness. Particularly with a group in which digitalization is highly variable, hybrid material enables analog and digital use simultaneously. For personal contact with medical professionals or social workers, the integration of digital formats such as (anonymous) online counseling should also be considered.

### Recommendations

**Figure 5:**
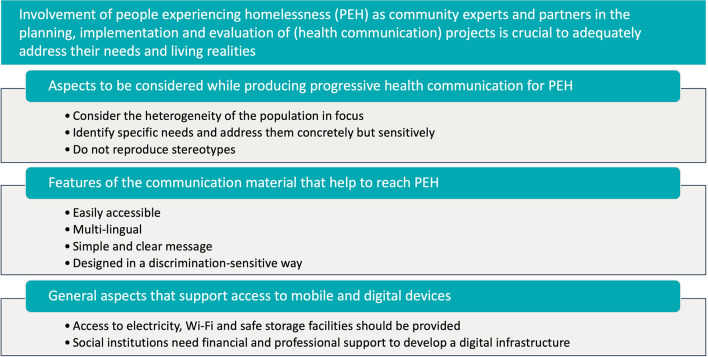
Key messages learned from this project.

Valuable lessons were learned through the project, that can help to strengthen participation of PEH and to consider their perspectives in health communication strategies (Figure 5).

Engaging marginalized populations as community experts and partners in the planning, implementation and evaluation of projects is crucial to adequately grasp their situation and needs. Strong links to the community, trust and the involvement of relevant stakeholders are indispensable when working with PEH.

Progressive health communication in terms of hybrid information material (analog and digital) considers the heterogeneity of the target group, identifies the specific needs and addresses them concretely but sensitively, without stereotypes. The material should be readily accessible, multi-lingual with a simple and clear message, addressing taboo topics while being designed in a discrimination-sensitive way. In this project people were addressed in their various spoken languages, including minority languages and a clear and simple language for functional illiterates. The experiences and feedback of peers and communities must be included in the development of information material.

Access to mobile and digital devices positively impacts the daily lives and health of PEH. Sustained use of the devices requires access to electricity and Wi-Fi as well as safe storage facilities. Social institutions should receive financial and professional support to develop a digital infrastructure and have it available for its users. Exclusion from (digital) information, on the other hand, is a major factor in the structural marginalization of PEH. Closing the digital gap can be a contribution to counteracting social and health inequalities.

## Data availability statement

The raw data supporting the conclusions of this article will be made available by the authors, without undue reservation.

## Ethics statement

The studies involving human participants were reviewed and approved by the Ethics Committee of the Charité – Universitätsmedizin Berlin (No.: EA2/168/21). The patients/participants provided their written informed consent to participate in this study. Written informed consent was obtained from the individual(s) for the publication of any potentially identifiable images or data included in this article.

## Author contributions

AS: conceptualization, methodology, investigation, and writing original draft. NS: conceptualization, methodology, and scientific advice. TL, TH, MH, and MW: methodology, investigation, and formal analysis. GE and JS: scientific advice and supervision. AL: project administration, conceptualization, and supervision. All authors: writing—review and editing. All authors contributed to the article and approved the submitted version.

## Funding

This study was funded by Bundesministerium für Bildung und Forschung/Federal Ministry of Education and Research: NaFoUniMedCovid19 (FKZ: 01KX2021) B-FAST. The funder had no role in the design and conduct of the study, analysis, interpretation of data, or in writing of the manuscript.

## Conflict of interest

The authors declare that the research was conducted in the absence of any commercial or financial relationships that could be construed as a potential conflict of interest.

## Publisher's note

All claims expressed in this article are solely those of the authors and do not necessarily represent those of their affiliated organizations, or those of the publisher, the editors and the reviewers. Any product that may be evaluated in this article, or claim that may be made by its manufacturer, is not guaranteed or endorsed by the publisher.

## Supplementary material

Supplementary Video 1 General information about COVID-19 (https://player.vimeo.com/video/510624249).

Supplementary Video 2 COVID-19: testing and quarantine (https://player.vimeo.com/video/510655463).
