# Supplementary material for: Participatory development and implementation of inclusive digital health communication on COVID-19 with homeless people
Source: Front Public Health. 2022 Nov 10;10:1042677. doi: 10.3389/fpubh.2022.1042677 (PMC9687377; doi:10.3389/fpubh.2022.1042677)
Supplement: Supplementary file 1 [file Data_Sheet_1.docx]

**Supplementary Interview guideline 1**. Guideline for semi-structured telephone-interviews for evaluation with institutions.

*Survey for facilities*

| How did you find out about the vaccination information **posters**? |  |
| --- | --- |
| Have you used the posters in your workplace? | ☐ Yes  ☐ No |
| **If Yes** | |
| How have the posters impacted on your work? |  |
| What were the reactions/comments from PEH, other facilities, colleagues, passers-by about the posters? |  |
| To what extent have conversations about vaccination occurred between clients  and/or employees as a result of the posters? |  |
| In your opinion, who or what was missing in the posters? |  |
| In general, what do you think about the use of posters for health information dissemination? |  |
| Which other topics could be addressed on future posters to support your work? |  |
| Which other topics could be addressed in future promotion material? |  |
| **If No** | |
| What were the reasons for not using them? |  |
| Do you have any suggestions for a format that could support you in health communication (possibly vaccination campaigns)? |  |
|  | |
| Are you familiar with the **videos** we made? | ☐ Yes  ☐ No |
| **If Yes** | |
| Have you been able to show the videos at your workplace? |  |
| In your opinion, who or what was missing in the videos? |  |
| Do you prefer digital health promotion material as opposed to posters? |  |
| Which health-related topics do you consider important as digital information for your work? |  |
| **If No** | |
| Do you prefer digital health promotion material as opposed to posters? |  |
| Which health-related topics do you consider important as digital information for your work? |  |
