# Supplementary material for: Participatory development and implementation of inclusive digital health communication on COVID-19 with homeless people
Source: Front Public Health. 2022 Nov 10;10:1042677. doi: 10.3389/fpubh.2022.1042677 (PMC9687377; doi:10.3389/fpubh.2022.1042677)
Supplement: Supplementary file 2 [file Data_Sheet_2.docx]

**Supplementary Interview guideline 2**. Guideline for semi-structured face-to-face-interviews for evaluation with PEH.

*Survey for PEH*

| Are you familiar with the Charité vaccination information **posters**? | ☐ Yes  ☐ No |
| --- | --- |
| **If Yes** | |
| Where have you seen the posters? |  |
| Which poster do you prefer?  What do you like/dislike about them? (motifs are demonstrated during the interview) |  |
| Did you feel addressed when you saw the posters? |  |
| What did you do/think when you saw the posters? |  |
| Did the poster inspire you to think about receiving a COVID-19 vaccination? |  |
| Did you then talk to other people about  to the COVID-19 vaccination? |  |
| Who or what would you suggest to be shown on the posters? |  |
| How do you feel about health information dissemination via posters? |  |
| **If No** (motifs are demonstrated during the interview) | |
| What do you like/dislike about it? |  |
| How do you feel about health information dissemination via posters? |  |
|  | |
| Are you familiar with the **videos** we made? | ☐ Yes  ☐ No |
| **If Yes** | |
| Where have you seen the videos? |  |
| What do you like/dislike about it? |  |
| What did you do/think when you saw the posters? |  |
| In your opinion, who or what is missing in the videos? |  |
| How do you feel about health information dissemination via videos? |  |
| **If No** | |
| Would you like to see the videos? |  |
| What is your spontaneous impression of these videos? |  |
