# Supplementary material for: Participatory development and implementation of inclusive digital health communication on COVID-19 with homeless people
Source: Front Public Health. 2022 Nov 10;10:1042677. doi: 10.3389/fpubh.2022.1042677 (PMC9687377; doi:10.3389/fpubh.2022.1042677)
Supplement: Supplementary file 3 [file Table_1.docx]

**Supplementary Table 1**. Cities with numbers and type of institutions across Germany that received the printed posters without or with request (from February 2021 to May 2022)

| **Type of institution/facility** |  | **Number** |
| --- | --- | --- |
| Facility for PEH | Clothing distribution facility | 6 |
|  | Day care center | 29 |
|  | Homeless consulting service | 21 |
|  | Hygiene facility | 3 |
|  | Medical facility | 14 |
|  | Night shelter | 11 |
|  | Soup kitchen | 8 |
|  | Facility for PEH (unspecified) | 54 |
| Facility for drug users |  | 9 |
| Facility for refugees |  | 2 |
| Municipality/Public facility |  | 6 |
| **Total** | | **163** |
|  |  |  |
| **Number of cities** |  | **53** |
| **Number of posters** |  | **1754** |

| **City** | **Type of institution** | **Category** | **Number of posters** |
| --- | --- | --- | --- |
| Alsfeld | Facility for PEH (unspecified) | Facility for PEH | 3 |
| Altentreptow | Facility for PEH (unspecified) | Facility for PEH | 14 |
| Augsburg | Facility for PEH (unspecified) | Facility for PEH | 4 |
| Bad Segeberg | Facility for PEH (unspecified) | Facility for PEH | 21 |
| Bergisch Gladbach | Facility for PEH (unspecified) | Facility for PEH | 64 |
| Berlin | Clothing facility | Facility for PEH | 7 |
| Berlin | Clothing facility | Facility for PEH | 7 |
| Berlin | Clothing facility | Facility for PEH | 7 |
| Berlin | Clothing facility | Facility for PEH | 7 |
| Berlin | Clothing facility | Facility for PEH | 7 |
| Berlin | Clothing facility | Facility for PEH | 7 |
| Berlin | Day care center | Facility for PEH | 7 |
| Berlin | Day care center | Facility for PEH | 7 |
| Berlin | Day care center | Facility for PEH | 7 |
| Berlin | Day care center | Facility for PEH | 7 |
| Berlin | Day care center | Facility for PEH | 7 |
| Berlin | Day care center | Facility for PEH | 7 |
| Berlin | Day care center | Facility for PEH | 7 |
| Berlin | Day care center | Facility for PEH | 7 |
| Berlin | Day care center | Facility for PEH | 7 |
| Berlin | Day care center | Facility for PEH | 7 |
| Berlin | Day care center | Facility for PEH | 7 |
| Berlin | Day care center | Facility for PEH | 7 |
| Berlin | Day care center | Facility for PEH | 7 |
| Berlin | Day care center | Facility for PEH | 7 |
| Berlin | Day care center | Facility for PEH | 7 |
| Berlin | Day care center | Facility for PEH | 7 |
| Berlin | Day care center | Facility for PEH | 7 |
| Berlin | Day care center | Facility for PEH | 7 |
| Berlin | Day care center | Facility for PEH | 7 |
| Berlin | Day care center | Facility for PEH | 7 |
| Berlin | Day care center | Facility for PEH | 7 |
| Berlin | Day care center | Facility for PEH | 7 |
| Berlin | Facility for drug users | Facility for drug users | 7 |
| Berlin | Facility for drug users | Facility for drug users | 7 |
| Berlin | Facility for drug users | Facility for drug users | 7 |
| Berlin | Facility for drug users | Facility for drug users | 7 |
| Berlin | Facility for drug users | Facility for drug users | 7 |
| Berlin | Facility for PEH (unspecified) | Facility for PEH | 7 |
| Berlin | Facility for PEH (unspecified) | Facility for PEH | 7 |
| Berlin | Facility for PEH (unspecified) | Facility for PEH | 7 |
| Berlin | Facility for PEH (unspecified) | Facility for PEH | 11 |
| Berlin | Facility for PEH (unspecified) | Facility for PEH | 21 |
| Berlin | Facility for PEH (unspecified) | Facility for PEH | 21 |
| Berlin | Facility for PEH (unspecified) | Facility for PEH | 14 |
| Berlin | Facility for PEH (unspecified) | Facility for PEH | 28 |
| Berlin | Homeless consulting service | Facility for PEH | 7 |
| Berlin | Homeless consulting service | Facility for PEH | 7 |
| Berlin | Homeless consulting service | Facility for PEH | 7 |
| Berlin | Homeless consulting service | Facility for PEH | 7 |
| Berlin | Homeless consulting service | Facility for PEH | 7 |
| Berlin | Homeless consulting service | Facility for PEH | 7 |
| Berlin | Homeless consulting service | Facility for PEH | 7 |
| Berlin | Homeless consulting service | Facility for PEH | 7 |
| Berlin | Homeless consulting service | Facility for PEH | 7 |
| Berlin | Homeless consulting service | Facility for PEH | 7 |
| Berlin | Homeless consulting service | Facility for PEH | 7 |
| Berlin | Homeless consulting service | Facility for PEH | 4 |
| Berlin | Hygiene facility | Facility for PEH | 7 |
| Berlin | Hygiene facility | Facility for PEH | 7 |
| Berlin | Hygiene facility | Facility for PEH | 7 |
| Berlin | Medical facility | Facility for PEH | 7 |
| Berlin | Medical facility | Facility for PEH | 7 |
| Berlin | Medical facility | Facility for PEH | 7 |
| Berlin | Medical facility | Facility for PEH | 7 |
| Berlin | Medical facility | Facility for PEH | 7 |
| Berlin | Medical facility | Facility for PEH | 7 |
| Berlin | Medical facility | Facility for PEH | 7 |
| Berlin | Medical facility | Facility for PEH | 7 |
| Berlin | Medical facility | Facility for PEH | 7 |
| Berlin | Medical facility | Facility for PEH | 7 |
| Berlin | Night shelter | Facility for PEH | 7 |
| Berlin | Night shelter | Facility for PEH | 7 |
| Berlin | Night shelter | Facility for PEH | 7 |
| Berlin | Night shelter | Facility for PEH | 7 |
| Berlin | Night shelter | Facility for PEH | 7 |
| Berlin | Night shelter | Facility for PEH | 15 |
| Berlin | Night shelter | Facility for PEH | 7 |
| Berlin | Night shelter | Facility for PEH | 7 |
| Berlin | Night shelter | Facility for PEH | 7 |
| Berlin | Night shelter | Facility for PEH | 7 |
| Berlin | Public facility | Municipality/Public Facility | 7 |
| Berlin | Soup kitchen | Facility for PEH | 7 |
| Berlin | Soup kitchen | Facility for PEH | 7 |
| Berlin | Soup kitchen | Facility for PEH | 7 |
| Berlin | Soup kitchen | Facility for PEH | 7 |
| Berlin | Soup kitchen | Facility for PEH | 7 |
| Berlin | Soup kitchen | Facility for PEH | 7 |
| Berlin | Soup kitchen | Facility for PEH | 7 |
| Berlin | Soup kitchen | Facility for PEH | 7 |
| Bochum | Facility for PEH (unspecified) | Facility for PEH | 28 |
| Bonn | Facility for PEH (unspecified) | Facility for PEH | 7 |
| Braunschweig | Facility for PEH (unspecified) | Facility for PEH | 28 |
| Braunschweig | Municipality | Municipality/Public Facility | 14 |
| Chemnitz | Facility for PEH (unspecified) | Facility for PEH | 7 |
| Cologne | Facility for PEH (unspecified) | Facility for PEH | 7 |
| Cologne | Facility for PEH (unspecified) | Facility for PEH | 3 |
| Darmstadt | Facility for refugees | Facility for refugees | 10 |
| Darmstadt | Homeless consulting service | Facility for PEH | 6 |
| Darmstadt | Facility for PEH (unspecified) | Facility for PEH | 14 |
| Duisburg | Facility for drug users | Facility for drug users | 20 |
| Duisburg | Facility for PEH (unspecified) | Facility for PEH | 1 |
| Duisburg | Facility for PEH (unspecified) | Facility for PEH | 21 |
| Düsseldorf | Facility for PEH (unspecified) | Facility for PEH | 14 |
| Düsseldorf | Facility for PEH (unspecified) | Facility for PEH | 28 |
| Essen | Facility for drug users | Facility for drug users | 14 |
| Essen | Facility for PEH (unspecified) | Facility for PEH | 35 |
| Essen | Medical facility | Facility for PEH | 7 |
| Frankfurt | Facility for PEH (unspecified) | Facility for PEH | 14 |
| Frankfurt | Municipality | Municipality/Public Facility | 13 |
| Frankfurt | Facility for PEH (unspecified) | Facility for PEH | 4 |
| Freiburg | Day care center | Facility for PEH | 28 |
| Giessen | Facility for PEH (unspecified) | Facility for PEH | 21 |
| Göppingen | Facility for PEH (unspecified) | Facility for PEH | 14 |
| Göttingen | Facility for PEH (unspecified) | Facility for PEH | 14 |
| Hamburg | Facility for PEH (unspecified) | Facility for PEH | 3 |
| Hamburg | Homeless consulting service | Facility for PEH | 7 |
| Hameln | Day care center | Facility for PEH | 28 |
| Hannover | Homeless consulting service | Facility for PEH | 53 |
| Hannover-Münden | Facility for PEH (unspecified) | Facility for PEH | 4 |
| Hattingen | Homeless consulting service | Facility for PEH | 7 |
| Herten | Homeless consulting service | Facility for PEH | 42 |
| Hildesheim | Day care center | Facility for PEH | 7 |
| Hof | Facility for PEH (unspecified) | Facility for PEH | 14 |
| Karlsruhe | Facility for PEH (unspecified) | Facility for PEH | 14 |
| Kassel | Day care center | Facility for PEH | 10 |
| Kassel | Facility for PEH (unspecified) | Facility for PEH | 4 |
| Koblenz | Facility for PEH (unspecified) | Facility for PEH | 7 |
| Krefeld | Facility for PEH (unspecified) | Facility for PEH | 14 |
| Lübeck | Facility for PEH (unspecified) | Facility for PEH | 14 |
| Ludwigsburg | Facility for PEH (unspecified) | Facility for PEH | 5 |
| Mainz | Medical facility | Facility for PEH | 7 |
| Mannheim | Facility for drug users | Facility for drug users | 10 |
| Mannheim | Facility for PEH (unspecified) | Facility for PEH | 14 |
| Mülheim an der Ruhr | Facility for PEH (unspecified) | Facility for PEH | 30 |
| Müllheim | Facility for PEH (unspecified) | Facility for PEH | 10 |
| München | Day care center | Facility for PEH | 7 |
| München | Facility for PEH (unspecified) | Facility for PEH | 4 |
| Neuss | Facility for PEH (unspecified) | Facility for PEH | 7 |
| Nienburg | Homeless consulting service | Facility for PEH | 8 |
| Nürnberg | Facility for PEH (unspecified) | Facility for PEH | 4 |
| Nürnberg | Medical facility | Facility for PEH | 12 |
| Regensburg | Facility for PEH (unspecified) | Facility for PEH | 21 |
| Saarbrücken | Day care center | Facility for PEH | 14 |
| Saarbrücken | Facility for PEH (unspecified) | Facility for PEH | 3 |
| Saarbrücken | Facility for PEH (unspecified) | Facility for PEH | 14 |
| Stuttgart | Facility for PEH (unspecified) | Facility for PEH | 15 |
| Stuttgart | Facility for PEH (unspecified) | Facility for PEH | 12 |
| Stuttgart | Facility for PEH (unspecified) | Facility for PEH | 14 |
| Stuttgart | Facility for refugees | Facility for refugees | 14 |
| Stuttgart | Homeless consulting service | Facility for PEH | 32 |
| Stuttgart | Public facility | Municipality/Public Facility | 3 |
| Uelzen | Homeless consulting service | Facility for PEH | 4 |
| Viersen | Medical facility | Municipality/Public Facility | 14 |
| Vogelsberg | Facility for PEH (unspecified) | Facility for PEH | 3 |
| Wesseling | Homeless consulting service | Facility for PEH | 7 |
| Wismar | Night shelter | Facility for PEH | 14 |
| Wolfsburg | Facility for PEH (unspecified) | Facility for PEH | 4 |
| Wuppertal | Municipality | Municipality/Public Facility | 50 |
| Würzburg | Day care center | Facility for PEH | 14 |
| Würzburg | Facility for drug users | Facility for drug users | 4 |
| Würzburg | Facility for PEH (unspecified) | Facility for PEH | 14 |
| **Total** |  |  | **1754** |
